# Luteolin Inhibits Breast Cancer Stemness and Enhances Chemosensitivity through the Nrf2-Mediated Pathway

**DOI:** 10.3390/molecules26216452

**Published:** 2021-10-26

**Authors:** Kuen-Jang Tsai, Hsin-Yi Tsai, Chin-Chuan Tsai, Tai-Yu Chen, Tsung-Hua Hsieh, Chun-Lin Chen, Lulekiwe Mbuyisa, Yaw-Bin Huang, Ming-Wei Lin

**Affiliations:** 1 Department of Surgery, E-Da Cancer Hospital, Kaohsiung 82445, Taiwan; tsai560612@gmail.com; 2 School of Medicine, College of Medicine, I-Shou University, Kaohsiung 82445, Taiwan; 3 School of Pharmacy, Kaohsiung Medical University, Kaohsiung 80708, Taiwan; y7952pipi@gmail.com; 4 Department of Chinese Medicine, E-Da Hospital, Kaohsiung 82445, Taiwan; ed103622@edah.org.tw (C.-C.T.); ed107076@edah.org.tw (T.-Y.C.); 5 The School of Chinese Medicine for Post-Baccalaureate, I-Shou University, Kaohsiung 82445, Taiwan; 6 Department of Medical Research, E-Da Hospital/E-Da Cancer Hospital, I-Shou University, Kaohsiung 82445, Taiwan; pelagice@yahoo.com.tw; 7 Department of Biological Science, National Sun Yat-sen University, Kaohsiung 80424, Taiwan; chunlinchen@mail.nsysu.edu.tw; 8 School of Medicine for International Students, College of Medicine, I-Shou University, Kaohsiung 82445, Taiwan; mlulekiwe@gmail.com; 9 Department of Nursing, College of Medicine, I-Shou University, Kaohsiung 82445, Taiwan; 10 Regenerative Medicine and Cell Therapy Research Center, Kaohsiung Medical University, Kaohsiung 80708, Taiwan

**Keywords:** luteolin, breast cancer, cancer stemness, chemosensitivity, Nrf2, Cripto-1

## Abstract

Cancer stem cells (CSCs) are subpopulations of tumor masses with unique abilities in self-renewal, stemness maintenance, drug resistance, and the promotion of cancer recurrence. Recent studies have suggested that breast CSCs play essential roles in chemoresistance. Therefore, new agents that selectively target such cells are urgently required. Reactive oxygen species (ROS)-producing enzymes are the reason for an elevated tumor oxidant status. The nuclear factor erythroid 2-related factor 2 (Nrf2) is a transcriptional factor, which upon detecting cellular oxidative stress, binds to the promoter region of antioxidant genes. By triggering a cytoprotective response, Nrf2 maintains cellular redox status. Cripto-1 participates in the self-renewal of CSCs. Herein, luteolin, a flavonoid found in *Taraxacum* *officinale* extract, was determined to inhibit the expressions of stemness-related transcriptional factors, the ATP-binding cassette transporter G2 (ABCG2), CD44, aldehyde dehydrogenase 1 activity as well as the sphere formation properties of breast CSCs. Furthermore, luteolin suppressed the protein expressions of Nrf2, heme oxygenase 1 (HO-1), and Cripto-1 which have been determined to contribute critically to CSC features. The combination of luteolin and the chemotherapeutic drug, Taxol, resulted in enhanced cytotoxicity to breast cancer cells. These findings suggest that luteolin treatment significantly attenuated the hallmarks of breast cancer stemness by downregulating Nrf2-mediated expressions. Luteolin constitutes a potential agent for use in cancer stemness-targeted breast cancer treatments.

## 1. Introduction

Chemotherapy is the treatment of choice for breast cancer, but available therapeutics have limited efficacy. Cancer stem cells (CSCs) are subpopulations of tumor masses with unique abilities in self-renewal, stemness maintenance, drug resistance, and the promotion of cancer recurrence [1,2,3]. Recent studies have suggested that CSCs play essential roles in chemoresistance [4]. In the context of breast cancer treatment, including the development of precision medical treatments, CSCs are a potential novel therapeutic target [5,6]. CSCs in breast cancers possess similar features to normal stem cells, which are involved in the production of antioxidants [7].

Nuclear factor-erythroid 2-related factor 2 (Nrf2) is the key transcription factor regulating oxidative homeostasis, which upon detecting cellular oxidative stress, binds to the promoter region of antioxidant genes to promote the expression of antioxidant enzymes. Moreover, Nrf2 is overexpressed in breast CSCs and is involved in CSCs’ survival as well as resistance to chemotherapy- or radiotherapy-induced oxidative stress in the tumor microenvironment [8,9]. Strategies targeting Nrf2 constitute promising candidates in cancers treatment [10,11]. Cancer cells contain higher levels of endogenous reactive oxygen species (ROS) than normal cells [12]. The high antioxidant capacity in CSCs keeps cellular ROS at a low level, which supports their stemness and contributes to CSCs’ survival and drug resistance [13].

Cripto-1 is expressed at high levels in various types of human tumors including colon, gastric, lung, and breast tumors [14,15,16,17]. Cripto-1 has also been identified in a small subset of stem-like cells in human malignant melanomas [18]. In esophageal squamous cell carcinoma, it acts as a functional marker of CSCs and predicts patient prognosis [19]. Furthermore, Cripto-1 regulates cell stemness and contributes to the etiology of triple-negative breast cancer [20]. Cripto-1 has been suggested to be a novel therapeutic target for triple-negative breast cancer [21,22].

The dandelion (*Taraxacum officinale*) is a well-known medicinal plant containing numerous polyphenolic flavonoids (including luteolin). Anti-inflammatory, antioxidant, and anticancer activity are among the numerous beneficial properties attributed to this plant [23,24]. Luteolin was reported to suppress brain, lung, prostate, pancreatic, colorectal, and breast cancer [25,26,27,28]. However, the mechanisms of stemness inhibition in breast cancer remain unclear. In this study, we evaluated the potential roles of luteolin in cancer stemness and chemoresistance inhibition in triple-negative breast cancer cells. We postulated that luteolin downregulated breast cancer stemness markers via the Nrf2-mediated pathway.

## 2. Results

### 2.1. Luteolin Inhibited Cancer Stemness Capacity in MDA-MB-231 Cells

**Figure 1:**
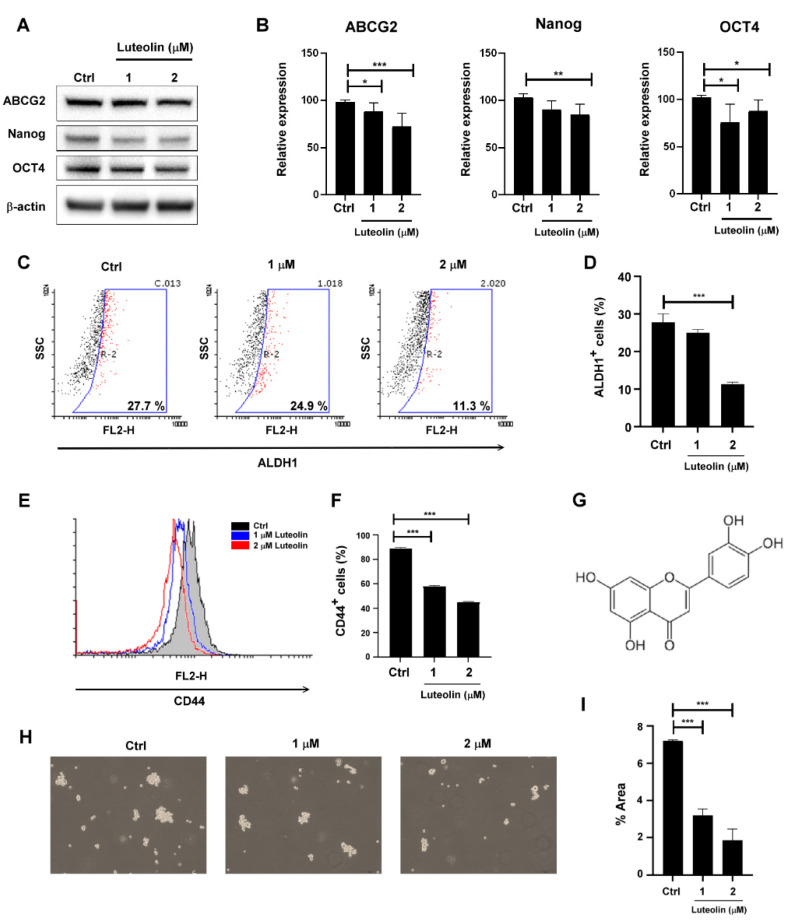
Stemness capacity in MDA-MB-231 cells was reduced by luteolin treatment: (**A**) cancer stem cell (CSC) marker proteins, such as ABCG2, Nanog, and Oct4, were analyzed through Western blotting after 48 h of luteolin treatment; (**B**) quantification of protein expression; (**C**) cells positive for aldehyde dehydrogenase 1 (ALDH1) were analyzed through flow cytometry after 48 h of luteolin treatment; (**D**) quantification of ALDH1^+^ cells; (**E**) CD44 expression was analyzed by flow cytometry after 48 h of luteolin treatment; (**F**) quantification of CD44 expression; (**G**) structural formula of luteolin; (**H**) images depicting the sphere formation ability following luteolin treatment; (**I**) quantification of the sphere formation area in MDA-MB-231 cells after luteolin treatment. Data are shown as the means ± standard errors of the mean of at least three independent experiments. Two-tailed Student’s *t*-test: * *p* < 0.05, ** *p* < 0.01, *** *p* < 0.001.

To determine the stemness characteristics of the MDA-MB-231 cells, the expression of stemness-related proteins (ABCG2, Nanog, and Oct4) were examined through Western blotting (Figure 1A,B). The activity of aldehyde dehydrogenase 1 (ALDH1) and stem cell biomarker CD44 was measured using flow cytometry (Figure 1C–E). CSC’s characteristics were evaluated using the sphere formation assay (Figure 1H–I). Treatment of MDA-MB-231 cells with various doses (from 1 to 2 μM) of luteolin for 48 h downregulated the stemness-related proteins (i.e., ABCG2, Nanog, Oct4, and CD44). In sum, the cells’ stemness capacity was impaired by luteolin treatment.

### 2.2. Luteolin Downregulated Antioxidant Proteins in Human MDA-MB-231 Cells

**Figure 2:**
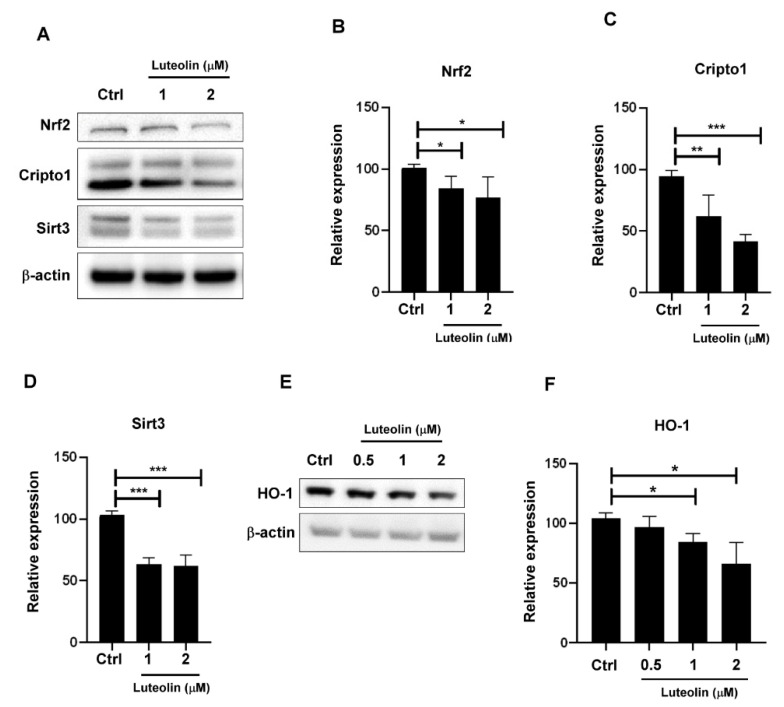
(**A**) Nrf2, Cripto-1, and SIRT3 expression was analyzed via Western blot after 48 h of luteolin treatment. (**B**–**F**) Quantification of Nrf2, Cripto-1, Sirt3, and HO-1 expression. Data are shown as the means ± standard errors of the mean of at least three independent experiments. Two-tailed Student’s *t*-test: * *p* < 0.05, ** *p* < 0.01, *** *p* < 0.001.

Nrf2 and Sirtuin 3 (Sirt3) expressions are correlated with redox imbalance in cancer cells [29]. These proteins regulate the expression of proteins protecting against oxidative damage. Cripto-1 acts as a functional marker of cancer stem cells [19]. Treatment of MDA-MB-231 cells with various doses (from 1 to 2 μM) of luteolin for 48 h downregulated Nrf2, sirt3, and Cripto-1 protein expressions (Figure 2A–D). Nrf2 was reported to act as a regulator in upregulating the expression of stress-response proteins, heme oxygenase-1 (HO-1) [30]. To confirm that luteolin play a role in the Nrf2/HO-1 axis, HO-1 expression was evaluated by Western blotting after luteolin treatment. As shown in Figure 2E,F, HO-1 protein expression was inhibited by luteolin treatment (from 0.5 μM to 2 μM) for 48 h. Our results demonstrated that luteolin suppressed Nrf2, HO-1, Sirt3, and Cripto-1 expression in MDA-MB-231 cells.

### 2.3. Nrf2 Regulated Stemness-Related Protein Expressions in MDA-MB-231 Cells

**Figure 3:**
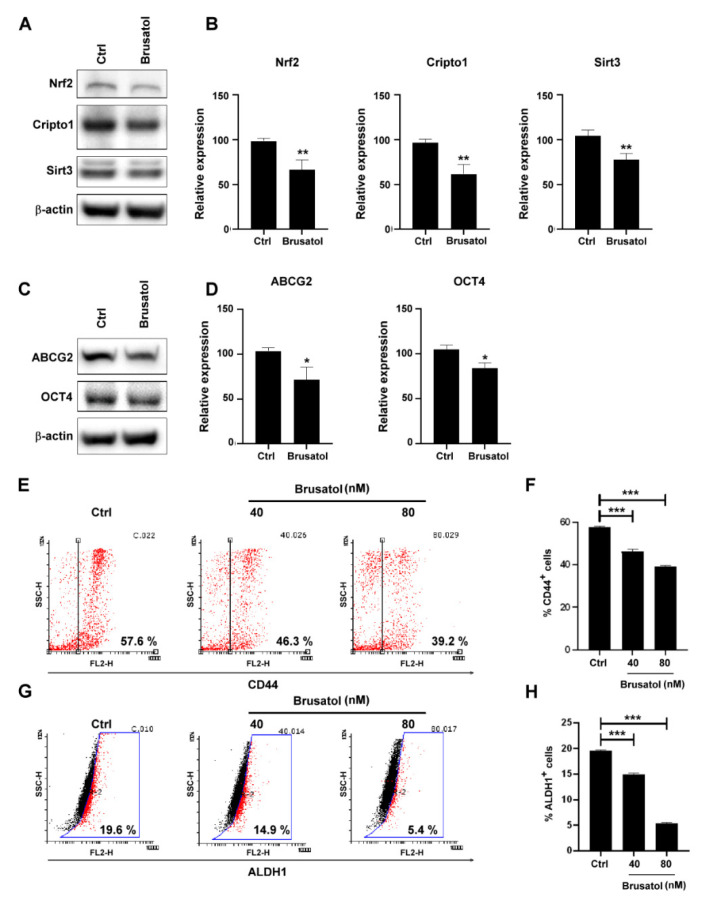
Brusatol regulated the expression of Cripto1, Sirt3, CD44, and ALDH1: (**A**) Nrf2, Cripto1, and SIRT3 expression was analyzed via Western blot after 48 h of brusatol treatment; (**B**) quantification of Nrf2, Cripto1, and Sirt3 expressions; (**C**) ABCG2 and OCT4 expressions were analyzed through Western blot after 48 h of brusatol treatment; (**D**) quantification of ABCG2 and OCT4 expression; (**E**) CD44 expression was analyzed through flow cytometry after 48 h of brusatol treatment; (**F**) quantification of CD44 expression; (**G**) ALDH1^+^ cells were analyzed through flow cytometry after 48 h of brusatol treatment; (**H**) quantification of ALDH1^+^ cells. Data are shown as the means ± standard errors of the mean of at least three independent experiments. Student’s *t*-test: * *p* < 0.05, ** *p* < 0.01, *** *p* < 0.001.

To determine whether Nrf2 or Cripto-1 plays a pivotal role in breast cancer stemness in MDA-MB-231 cells, the Nrf2 inhibitor, brusatol, was used to evaluate the expression of Cripto-1 and the other stemness-related proteins. As presented in Figure 3, treatment with brusatol (40 nM) for 48 h yielded similar results to those obtained with luteolin treatment. Specifically, brusatol suppressed the expression of ABCG2, CD44, Oct4, Sirt3, ALDH1, and also Cripto-1.

### 2.4. Luteolin Regulated Breast Cancer Stemness via the Nrf2-Mediated Pathway

**Figure 4:**
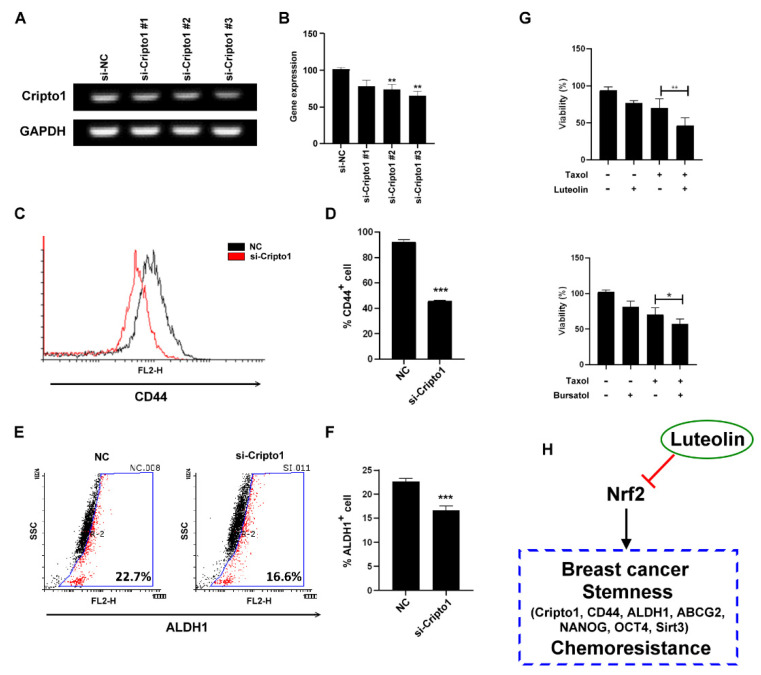
Luteolin inhibited stemness and chemoresistance via Cripto-1 expression: (**A**) a reverse transcription–polymerase chain reaction was conducted to determine the level of Cripto-1 mRNA in cells transfected with Cripto1 small-interfering RNA; (**B**) quantification of *Cripto-1* expression in MDA-MB-231 cells; (**C**) CD44 expression in MDA-MB231 cells was analyzed through flow cytometry after *Cripto-1* knockdown; (**D**) quantification of CD44 expression; (**E**) ALDH1^+^ expression in MDA-MB-231 cells was analyzed by flow cytometry after *Cripto-1* knockdown; (**F**) quantification of ALDH1^+^ cells; (**G**) cell viability of MDA-MB-231 subjected to combined treatment with 1 μM luteolin and 40 nM Taxol for 48 h; (**H**) summary of the mechanisms by which luteolin inhibits triple-negative breast cancer stemness. Student’s *t*-test: * *p* < 0.05, ** *p* < 0.01, *** *p* < 0.001.

Cripto-1 acts as a functional marker of CSCs [19]. We further used Cripto-1 siRNA to determine whether Cripto-1 regulated stemness-related protein expression in MDA-MB-231 cells. To test which siRNA could potently inhibit Cripto-1 expression, RT-PCR was used to evaluate Cripto-1 mRNA expression. As shown in Figure 4A,B, si-Cripto-1#3 potently inhibited Cripto-1 mRNA expression. Knockdown of Cripto-1 by si-Cripto-1#3 downregulated CD44 and ALDH1 (Figure 4C–F). On the basis of the observation that the inhibition of Nrf2 by luteolin or brusatol downregulated Cripto-1 expression, we treated MDA-MB-231 cells with a combination of the clinical chemotherapy drug, Taxol (paclitaxel), with either luteolin or brusatol to enhance the cytotoxicity to MDA-MB-231 cells (Figure 4G). Luteolin regulated the breast cancer stemness and chemoresistance by the Nrf2-mediated pathway.

## 3. Discussion

CSCs are defined as a small subset of cells within the tumor microenvironment with intrinsic capabilities of self-renewal and differentiation. Failure of cancer treatment involving tumor recurrence has been ascribed to the existence of these cells [31]. Therefore, targeting CSCs for further therapeutic strategy is important. Signaling molecules involved in the maintenance of CSCs include Nrf2 and Cripto-1. Nrf2 regulates stem cell differentiation by directly binding to upstream regions of pluripotency genes *Oct4* and *Nanog* to promote their expression [8]. Given that CD44^+^/CD24^−^ is the phenotype of breast CSCs [32], Nrf2 levels could also be a prognostic marker in breast cancer [33,34]. Brusatol, which inhibits Nrf2 by enhancing protein ubiquitination [35], downregulated similar stemness-related proteins including CD44. This suggests that luteolin acted as an Nrf2 inhibitor to suppress breast cancer stemness in the present study.

Cripto-1 promotes epithelial–mesenchymal transition (EMT) in mammary tumors in mice [36]. This might be associated with the EMT gene expression program of CSCs to support their abilities in self-renewal, invasion, and metastasis. In one study, the stemness-related transcription factors, Nanog and Oct4, modulated Cripto-1 expression [37]. In another study, Cripto-1 was found to be essential for triggering and maintaining the expression of Nanog and Oct4 [38]. These results indicate the presence of a positive feedback regulatory network of Cripto-1-mediated stemness signaling. The downregulation of Cripto-1 by luteolin or Nrf2 inhibitor, brusatol, suggests that luteolin inhibits breast cancer stemness through the Nrf2-mediated pathway. However, in the present study, luteolin may be not a specific inhibitor for Nrf2 or Cripto-1. It may inhibit Nrf2 and Cripto-1 indirectly. The possibility that luteolin inhibits Nrf2 and Cripto-1 by parallel signal pathways could not be excluded. Some studies reported that luteolin activates the Nrf2 signaling pathway [39,40]; however, luteolin-induced Nrf2 inhibition was also found in previous studies. Chian et al. reported that luteolin is a strong inhibitor of Nrf2 in vitro. They further confirmed that luteolin inhibited the Nrf2 signaling pathway and lung tumor growth in vivo by using an Nrf2^−/−^ mouse model [41,42,43,44]. The effects of luteolin on the Nrf2 pathway may be cell-type specific and involve multiple signaling pathways and mechanisms.

ALDH1 is a CSC marker, and high ALDH1 expression has been observed to facilitate tumor growth and be related to drug resistance [45,46]. In one study, the increase in ALDH1 was associated with Nrf2 upregulation [47]. In another investigation, the silencing of Nrf2 reduced the expression of ALDH1 in pancreatic cancer cells and promoted their sensitivity to the chemotherapeutic drug, 5-fluorouracil [48]. Furthermore, the Nrf2 pathway was reported to regulate ALDH1 and contribute to radioresistance in breast CSCs [49]. In the present study, luteolin reduced ALDH1^+^ breast cancer cells thorough the downregulation of Nrf2.

Sirt3 is an NAD^+^ dependent deacetylase that resides primarily in mitochondria and functions to maintain mitochondrial homeostasis under stress. In one study, Sirt3^−/−^ cells reduced the expression of antioxidant enzymes such as MnSOD [50]. Another study noted that Sirt3 reduces ROS production in mitochondria within glioma stem cells [51]. This metabolic modulation facilitated adaptation to stress and maintained stemness in CSCs. The deactivation of SIRT3 led to metabolic alterations, loss of stemness, and suppression of tumor formation in glioma stem cells in vivo. In the present study, the inhibition of Sirt3 by luteolin or brusatol suggested that luteolin suppressed Sirt3 expression in breast cancer cells to attenuate stress adaptation and chemoresistance by reducing the expression of antioxidant enzymes.

Targeting CSCs and combined chemotherapies potentially prevent cancer recurrence [52]. Luteolin has been used in traditional Chinese medicine for treating various diseases, especially cancer [26]. It increases levels of intracellular ROS by activating a lethal stress response in the endoplasmic reticulum and inducing mitochondrial dysfunction [53]. Herein, luteolin downregulated the expression of stemness-related proteins in the MDA-MB-231 cells and enhanced their chemosensitivity. The findings suggest that luteolin treatment significantly attenuates the hallmarks of breast cancer stemness by downregulating Nrf2 expression. Thus, luteolin can potentially be used in stemness-targeted breast cancer treatments.

## 4. Materials and Methods

### 4.1. Cell Culture and Reagent

The human triple-negative breast cancer cell line MDA-MB-231 was obtained from the Bioresource Collection and Research Center (Hsinchu, Taiwan) and cultured in Leibovitz’s L-15 medium (Gibco, Waltham, MA, USA) containing 10% fetal bovine serum (Gibco, Waltham, MA, USA) and stabilized under 5% CO_2_ at 37 °C. Luteolin, brusatol, and the chemotherapeutic Taxol were purchased from Sigma-Aldrich (St. Louis, MO, USA).

### 4.2. Cell Viability Analysis

Cells were seeded in 96-well plates in quadruplicate (6000 cells/well) and contained 200 μL of medium for 24 h before treatment. Next, cells were treated with 2 μM luteolin or 40 nM brusatol combined with 10 nM paclitaxel or not for 48 h. The cell viability was analyzed using Cell Counting Kit-8 (Sigma–Aldrich, St. Louis, MO, USA). After treatment, 10 μL of CCK-8 solution was added to each well, and the plate was incubated at 37 °C for 2 h. Finally, absorbance was measured at 450 nm on a microplate reader (Bio-Rad, Hercules, CA, USA).

### 4.3. Flow Cytometry Analysis

After treatment, cells were washed with cold phosphate-buffered saline and stained with surface marker CD44 (BD Biosciences, San Jose, CA, USA) before flow cytometry analysis. For ALDH1 activity evaluation, cells were stained using the AldeRed ALDH Detection Assay kit (Sigma–Aldrich, St. Louis, MO, USA) and analyzed through flow cytometry.

### 4.4. Western Blotting

The total protein was extracted, and equal quantities of total protein were separated using sodium dodecyl sulfate–polyacrylamide gel electrophoresis and transferred onto polyvinylidene fluoride membranes. Membranes were blocked with blocking buffer (Bio-Rad, Hercules, CA, USA) at room temperature and incubated with primary antibodies at 4 °C after washing with PBST. Membranes were developed using an electrochemiluminescence detection system after incubation with secondary antibodies.

### 4.5. siRNA Transfection Assay

Small-interfering RNAs (siRNAs) were purchased from Thermo Fisher Scientific. Cells were transfected with siRNA duplexes using the jetPRIME Versatile DNA/siRNA transfection reagent (Polyplus, New York, NY, USA) according to the manufacturer’s instructions. The sense and antisense strands of Cripto-1 siRNA were as follows: si-Cripto1#1: 5′-GGA UCA UGG CCA UUU CUA AAG UCU U-3′ (sense) and 5′-AAG ACU UUA GAA AUG GCC AUG AUC C-3′ (antisense); si-Cripto1#2: 5′-UCA UGC AAA UUU CAU GAC CAG UAA A-3′ (sense) and 5′-UUU ACU GGU CAU GAA AUU UGC AUG A-3′ (antisense); si-Cripto1#3: 5′-GGG CCA UCA GGA AUU UGC UCG UCC A-3′ (sense) and 5′-UGG ACG AGC AAA UUC CUG AUG GCC C-3′ (antisense).

### 4.6. Reverse Transcription-Polymerase Chain Reaction (RT-PCR)

Total RNA was extracted using the Quick-RNA Miniprep kit (Zymo Research, Irvine, CA, USA). For complementary DNA synthesis, 500 ng of total RNA was reverse transcribed using the GoTaq 1-Step RT-qPCR System (Promega, Madison, WI, USA) according to the manufacturer’s instructions. Regarding PCR reaction, Cripto-1 and GAPDH transcripts were amplified with GoTaq^®^ Green Master Mix (Promega, Madison, WI, USA). The primer sequences were as follows: Cripto-1: forward: 5′-GATACAGCACAGTAAGGAGC-3′ and reverse: 5′-TAGTTCTGGAGTCCTGGAAG-3′; GAPDH: forward: 5′-AGATGATGACCCTTTTGGCTC-3′ and reverse: 5′-AAGGTCGGAGTCAACGGATTT-3′.

### 4.7. Sphere Formation Analysis

For the sphere formation assay, MDA-MB-231 cells were seeded in low-adhesion plates with serum-free Leibovitz’s L-15 medium (Gibco, Waltham, MA, USA) containing 20 ng/mL EGF, 20 ng/mL FGF, and 2% B27 (Gibco, Waltham, MA, USA) and treated with luteolin (1 or 2 μM) for 7 days, before being examined using ImageJ software.

### 4.8. Statistical Analysis

Data are presented as means ± standard deviations. The Student’s *t*-test was conducted for between-group comparisons. Differences were considered statistically significant at least at *p* < 0.05.

## 5. Conclusions

The findings suggest that luteolin treatment significantly attenuated the hallmarks of breast cancer stemness by downregulating Nrf2-mediated expression. Thus, luteolin can potentially be used in stemness-targeted breast cancer treatments.

## Data Availability

All data sets generated or analyzed in this study were included in the published article. Detailed data sets supporting the current study are available from the corresponding author upon request. This study did not generate new codes.

## Sample Availability

Samples of the compounds are available from the authors.
